# Supplementary material for: Modelling Chlamydia and HPV co-infection in patient-derived ectocervix organoids reveals distinct cellular reprogramming
Source: Nat Commun. 2022 Feb 24;13:1030. doi: 10.1038/s41467-022-28569-1 (PMC8873204; doi:10.1038/s41467-022-28569-1)
Supplement: Supplementary file 1 — Supplementary Information [file 41467_2022_28569_MOESM1_ESM.pdf]

Modelling Chlamydia and HPV co-infection in patient-derived ectocervix organoids reveals distinct cellular reprogramming

Koster S, Gurumurthy RK et al.

Supplementary Figures and Legends

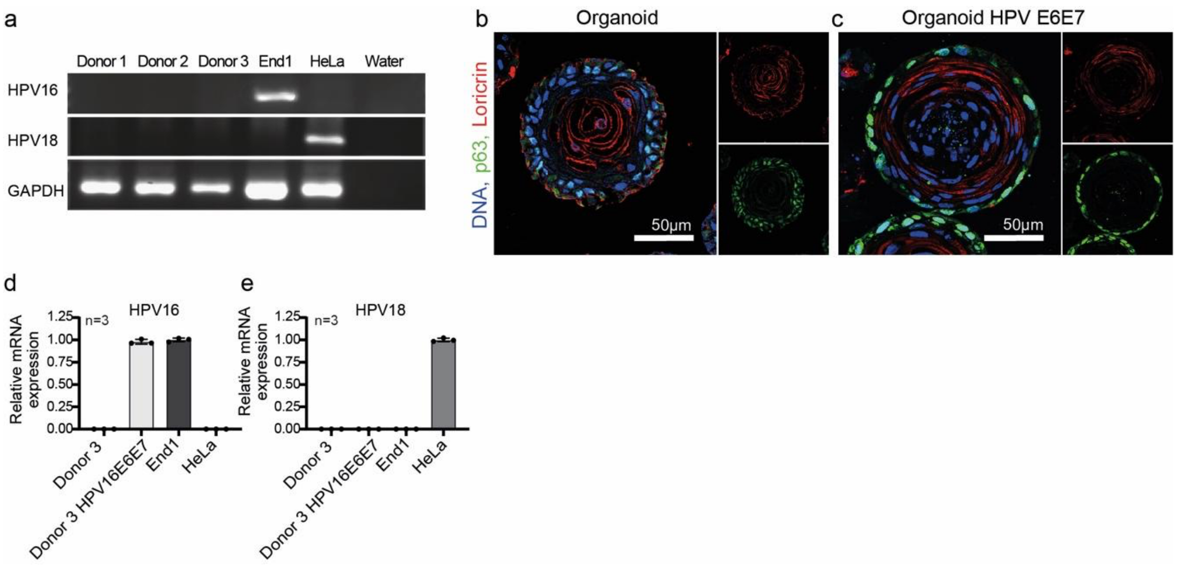

Supplementary Fig. 1: Ectocervical organoids expressing HPV16 E6E7 enhance organoid growth. (a) DNA isolated from ectocervical organoids from healthy donors was subjected to PCR to detect HPV16 and HPV18 E6E7*.* HeLa and End 1 cells were used as positive controls for HPV18 and HPV16, respectively. Data represent three biological replicates. (b-c) Representative confocal images of human ectocervical organoids without (b) and with (c) HPV16 E6E7 expression immunolabeled for p63 and Loricrin, Nuclei are shown in blue. Images represent three biologically independent experiments. (d-e) Relative mRNA expression of HPV16 E6E7 (d) and HPV18 E6E7 (e) genes analyzed by qRT-PCR. Data represented as mean ± SD of results normalized to mRNA expression of positive controls End1 and HeLa cells, respectively. Source data are provided as a Source Data file.

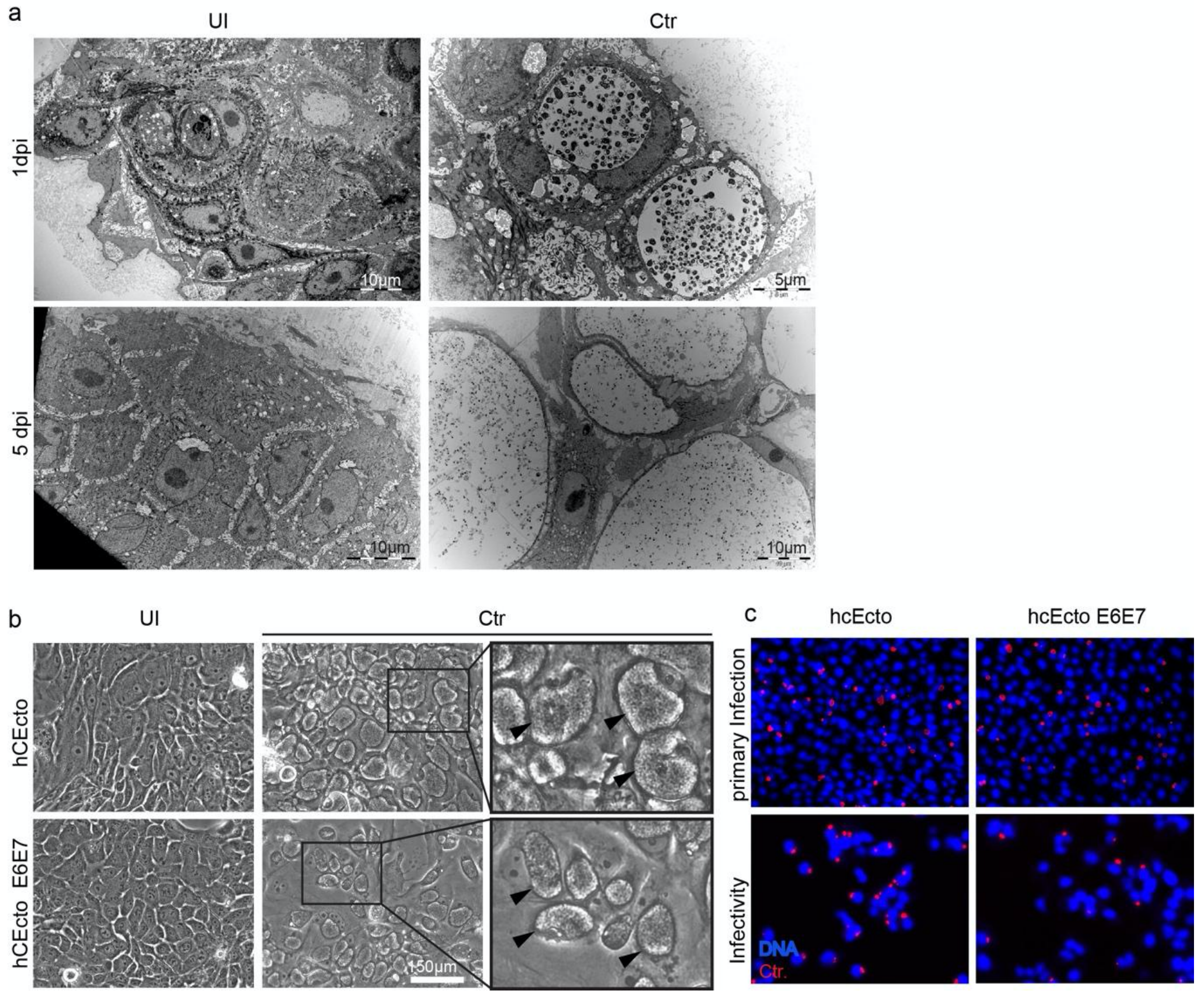

Supplementary Fig. 2: Ectocervical organoids as models for *C. trachomatis* and HPV co-infection studies. (a) Transmission electron micrographs of hCEcto organoids at 1 and 5dp Ctr infection. (b) Representative phase-contrast images of uninfected (UI) and Ctr-infected hCEcto and hCEcto E6E7 2D stem cells at 48hpi, arrowheads in inserts show inclusions. (c) Representative fluorescent images of Ctr primary infection and infectivity of hCEcto and hCEcto E6E7 cells. Images were taken with an automated microscope at 10x magnification. (a-c) Shown are representative images from three biological replicates.

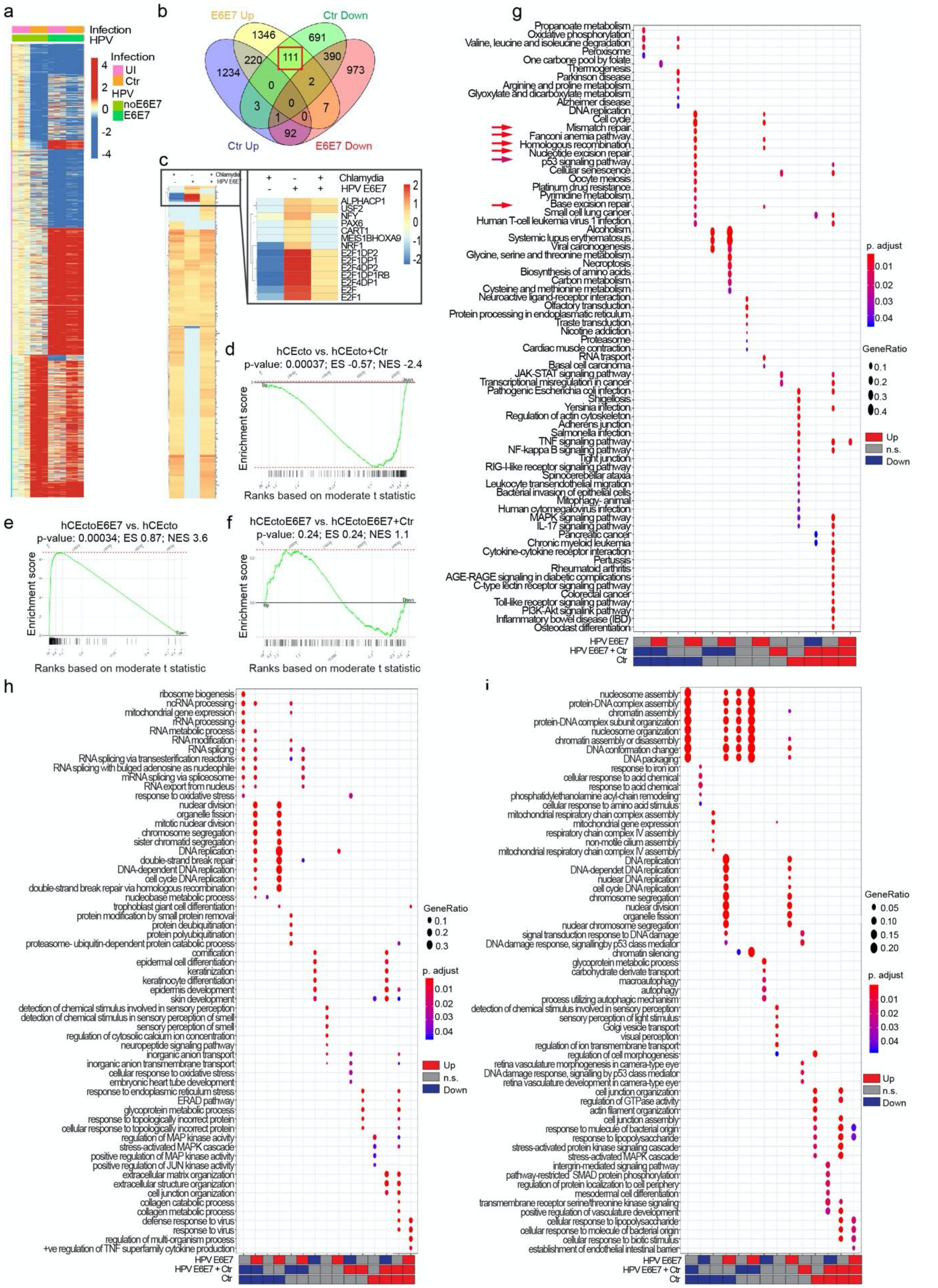

Supplementary Fig. 3: Host transcriptional response in human ectocervical organoids of C. trachomatis, HPV16 E6E7, and co-infection. (a) Heatmap of DEG in hCEcto and

hCEcto E6E7 organoids with or without Ctr infection for 5d with p-value< 0.05; log2 FC >1 or < -1 between any of the conditions from three replicates. The color bar depicts expression values after subtracting the mean of uninfected hCEcto samples and dividing by SD of each probe. (b) Venn diagram comparing genes significantly (log2 FC ≥ 2, p-value<0.05) up or down-regulated after Ctr infection or HPV E6E7 expression in hCEcto organoids. The red box highlights genes upregulated by HPV E6E7 and downregulated by Ctr. (c) Heatmap showing GSEA enrichment (–log10(p-value)) of genes that share Cis-regulatory motifs for transcription factors (stated in the right column). (d-f) GSEA analysis on DEG comparisons of hCEcto vs hCEcto+Ctr (d), hCEcto E6E7 vs hCEcto (e), hCEcto E6E7 vs hCEcto E6E7+Ctr (f) organoids. (g) Dot plot showing significantly up or down-regulated KEGG pathways among the DEG from hCEcto and hCEcto E6E7 organoids with or without Ctr infection. Red arrows highlight DNA repair pathways. Dot diameter refers to the gene ratio within a group. The fill color depicts the adjusted p-value. (h-i) GO terms associated with DEG from hCEcto and hCEcto E6E7 with or without Ctr infection from 2D stem cells (h) and 3D organoids (i).

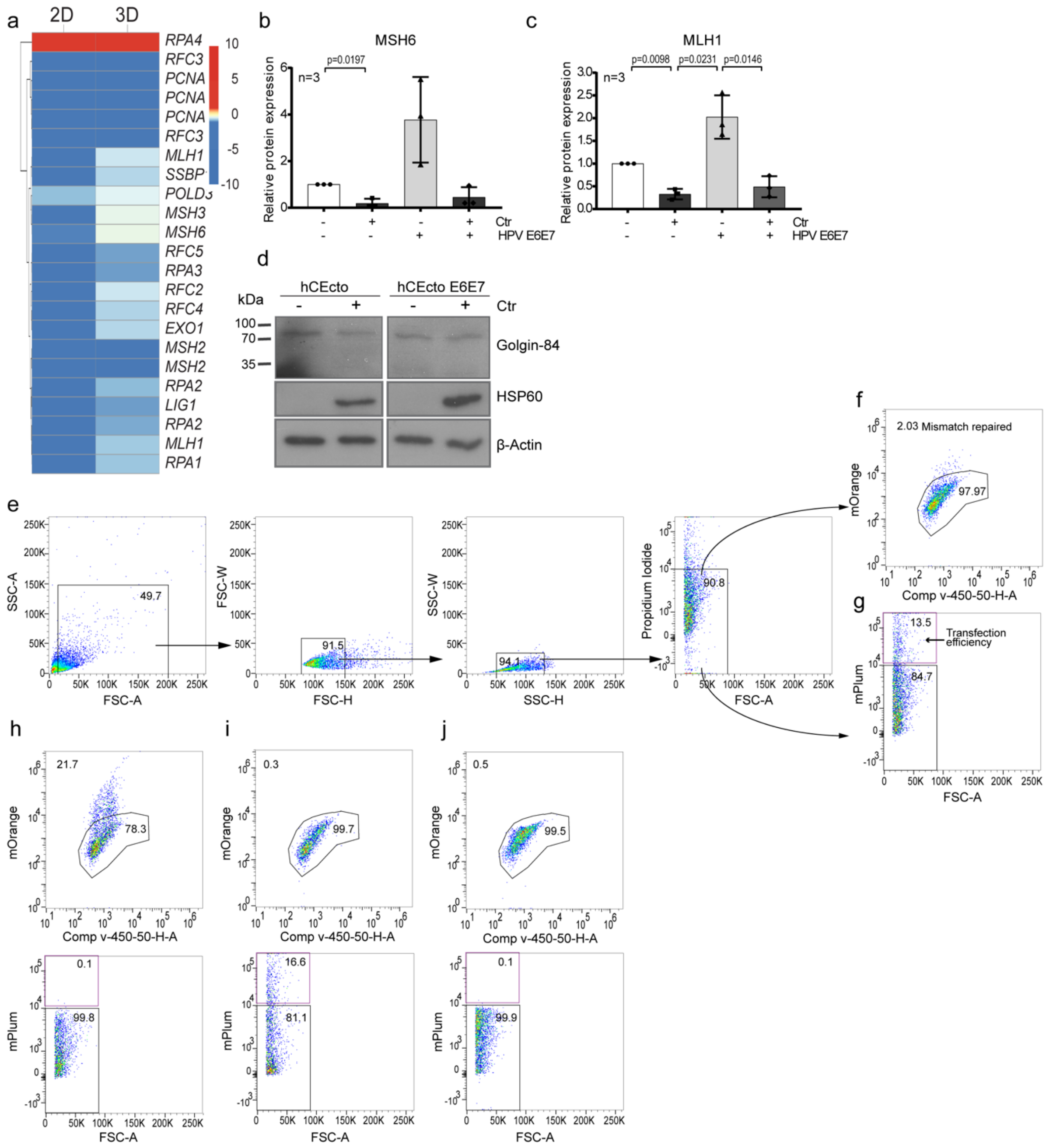

Supplementary Fig. 4: Chlamydia suppresses MMR in human ectocervical 2D stem cells and organoids. (a) Heatmap depicting the differential expression of MMR pathway genes from hCEcto 2D stem cells and organoids infected with Ctr for 48h or 5d, respectively. The color bar depicts log2 FC. (b-c) Shown are the densitometry quantification of MSH6 (b) and MLH1 (c) from immunoblots shown in Figure 4f. Densitometry values for MSH6 and MLH1 immunoblots were normalized to the β-actin values, and data representing the relative FC compared to an uninfected control are shown. Shown is the mean ± SD. Statistical significance was calculated using two-sided t-test, P-values are indicated. (d) hCEcto and hCEcto E6E7 2D stem cells were infected with Ctr for 48 h, and cell lysates were subjected to immunoblot analysis for indicated proteins and Chlamydia HSP60 and β-actin as a loading

control. Data are representative of three independent experiments. kDa, kilo Dalton. (e-j) Gating strategy related to FACS data in Figure 4j-k. (e) Sequential gating for elimination of cell debris, doublets, aggregates and dead cells. (f) Gating for the mOrange positive Mismatch repaired cell population (2%). (g) Determination of the transfection efficiency by identifying the mPlum positive cell population (13.5%; purple box). (h-j) Control reporter plasmids expressing the fluorescent proteins mOrange or mPlum were used to set the gating for (h) mOrange, (i) mPlum positive cells and (j) negative control without any plasmid. Source data are provided as a Source Data file.

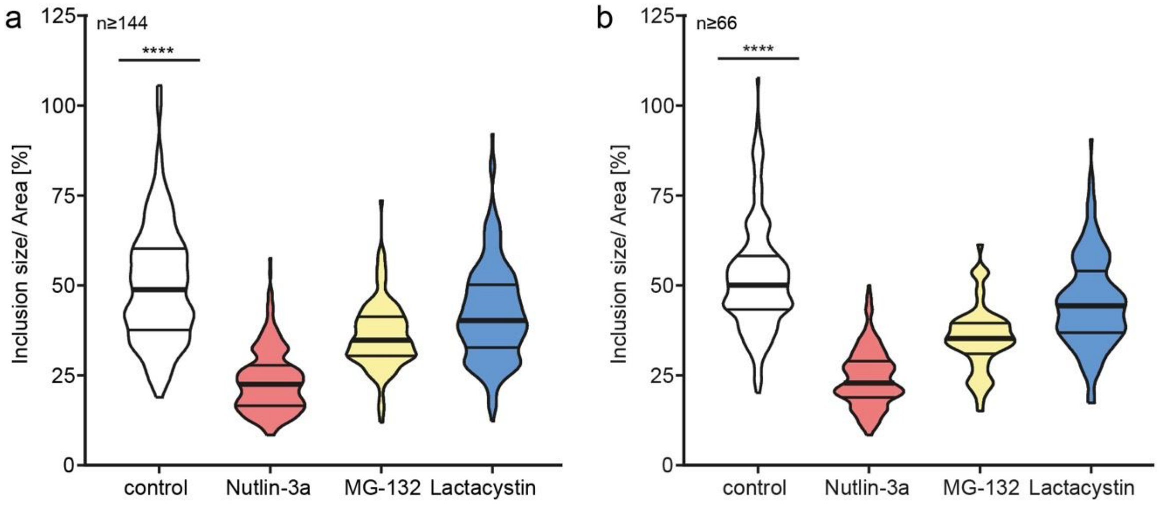

Supplementary Fig. 5: Impact of inhibition of proteasomal degradation and p53-MDM2 axis on *C. trachomatis* development. hCEcto and hCEcto E6E7 2D stem cells were infected with Ctr for 48h with or without additional treatment with Nutlin-3a (10 µM) (red) or Lactacystin (10 µM) (blue) from 2hpi or 24hpi with MG-132 (5 µM) (yellow). Quantification of the inclusion size by ImageJ from hCEcto (a) and hCEcto E6E7 (b). Representative data of two biological replicates are presented as violin plots min–max, lines are 25 and 75th percentile, bold line is median. Statistical significance was calculated using one-way ANOVA, P-values are indicated. Source data are provided as a Source Data file.

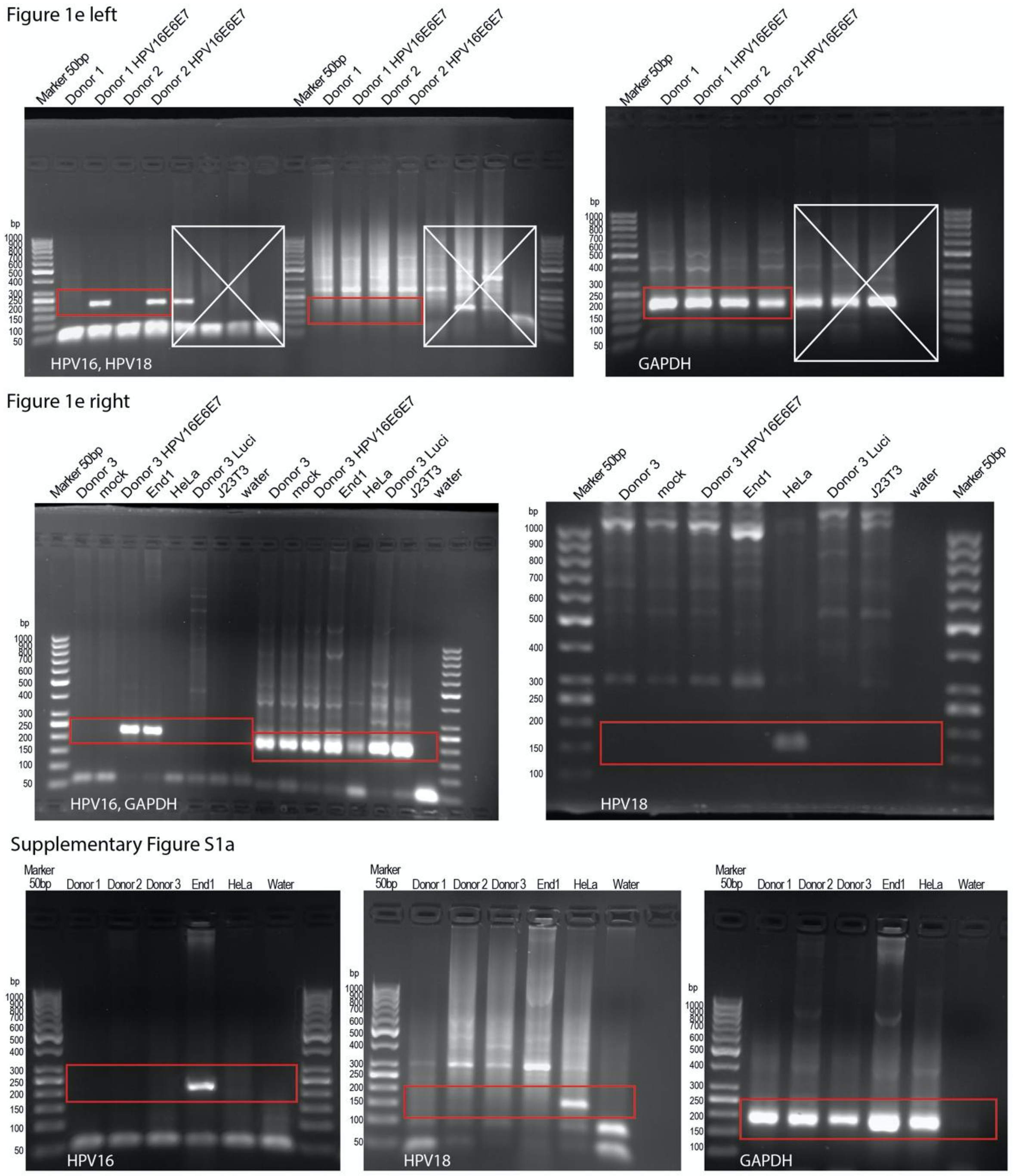

Supplementary Fig. 6: Uncropped scans of PCR gels blots. Molecular weights are in bp.

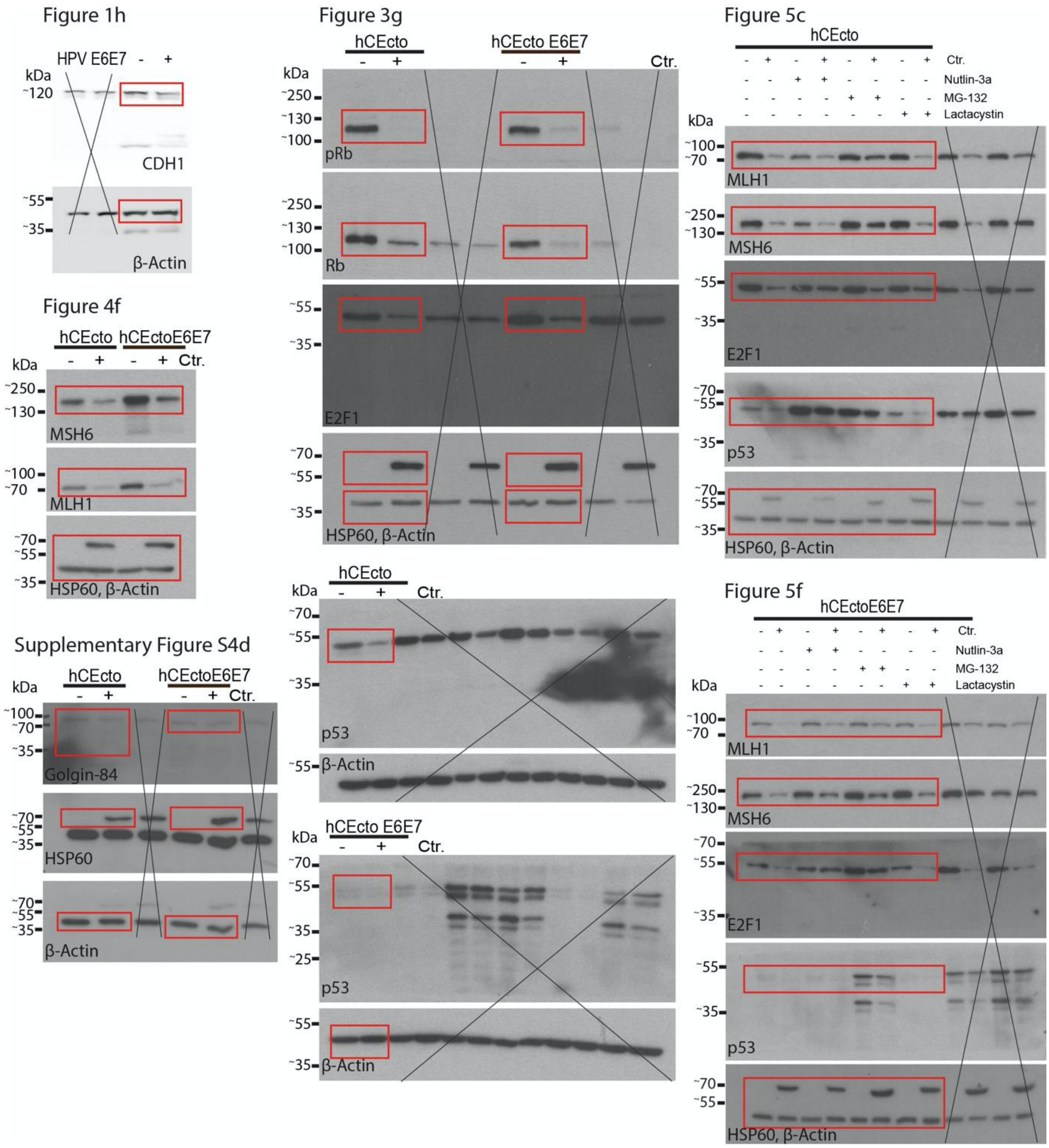

Supplementary Fig. 7: Uncropped scans of Western blots. Molecular weights are in kDa.
