# Supplementary material for: Modelling Chlamydia and HPV co-infection in patient-derived ectocervix organoids reveals distinct cellular reprogramming
Source: Nat Commun. 2022 Feb 24;13:1030. doi: 10.1038/s41467-022-28569-1 (PMC8873204; doi:10.1038/s41467-022-28569-1)
Supplement: Supplementary file 3 — Description of Additional Supplementary Files [file 41467_2022_28569_MOESM3_ESM.pdf]

Description of Additional Supplementary Files

File Name: Supplementary Data 1

Description: Contains information about differentially expressed genes from hCEcto and hCEcto E6E7 with or without *Chlamydia trachomatis* infection comparisons in 2D Stem cells and 3D organoids.

File Name: Supplementary Data 2

Description: Contains information about genes representing different groups of the Venn diagram in 2D stem cells and 3D organoids.

File Name: Supplementary Data 3

Description: Contains information about list of enriched transcription factors in 2D stem cells and 3D organoids.

File Name: Supplementary Data 4

Description: Contains information about gene ontology (GO) terms associated with differentially expressed genes from hCEcto and hCEcto E6E7 2D stem cell or 3D organoids with or without Chlamydia trachomatis infection.

File Name: Supplementary Data 5

Description: Contains information about Up or down-regulated KEGG pathways among the differentially expressed genes from hCEcto and hCEcto E6E7 2D stem cell or 3D organoids with or without Chlamydia trachomatis infection.

File Name: Supplementary Data 6

Description: Contains information about Fisher dream target genes.

File Name: Supplementary Data 7

Description: Contains information about E2F target genes.
