# Supplementary material for: Modelling Chlamydia and HPV co-infection in patient-derived ectocervix organoids reveals distinct cellular reprogramming
Source: Nat Commun. 2022 Feb 24;13:1030. doi: 10.1038/s41467-022-28569-1 (PMC8873204; doi:10.1038/s41467-022-28569-1)
Supplement: Supplementary file 11 — Reporting Summary [file 41467_2022_28569_MOESM11_ESM.pdf]

nature research

Corresponding author(s): Dr Cindrilla Chumduri

Last updated by author(s): 18-01-2022

Reporting Summary

Nature Research wishes to improve the reproducibility of the work that we publish. This form provides structure for consistency and transparency in reporting. For further information on Nature Research policies, see our Editorial Policies and the Editorial Policy Checklist.

Statistics

For all statistical analyses, confirm that the following items are present in the figure legend, table legend, main text, or Methods section.

n/a	Confirmed	
☐	☒	The exact sample size (n) for each experimental group/condition, given as a discrete number and unit of measurement
☐	☒	A statement on whether measurements were taken from distinct samples or whether the same sample was measured repeatedly
☐	☒	The statistical test(s) used AND whether they are one- or two-sided<br>*Only common tests should be described solely by name; describe more complex techniques in the Methods section.*
☒	☐	A description of all covariates tested
☐	☒	A description of any assumptions or corrections, such as tests of normality and adjustment for multiple comparisons
☐	☒	A full description of the statistical parameters including central tendency (e.g. means) or other basic estimates (e.g. regression coefficient) AND variation (e.g. standard deviation) or associated estimates of uncertainty (e.g. confidence intervals)
☐	☒	For null hypothesis testing, the test statistic (e.g. F, t, r) with confidence intervals, effect sizes, degrees of freedom and P value noted<br>*Give P values as exact values whenever suitable.*
☒	☐	For Bayesian analysis, information on the choice of priors and Markov chain Monte Carlo settings
☒	☐	For hierarchical and complex designs, identification of the appropriate level for tests and full reporting of outcomes
☒	☐	Estimates of effect sizes (e.g. Cohen's d, Pearson's r), indicating how they were calculated

Our web collection on statistics for biologists contains articles on many of the points above.

Software and code

Policy information about availability of computer code

Data collection	Microarray image data were analyzed and extracted with the Image Analysis/Feature Extraction software G2567AA v. A.11.5.1.1 (Agilent Technologies). FACSDiva software (BDbiosciences) V8.0.1, StepOneTM Software (v2.3, Applied Biosystems)
Data analysis	Graphpad Prism version 8 was used for statistical analysis.<br>Gene set enrichment was performed using the GSEA software from<br>http://software.broadinstitute.org/gsea/downloads.jsp.<br>R-3.3 was obtained from https://cran.r-project.org/<br>Adobe Photoshop and illustrator version v23.1<br>ImageJ v1.51f<br>FlowJo V10 (FlowJo, LLC)<br>VENNY 2.1 [http://bioinfogp.cnb.csic.es/tools/venny/]<br>ScanR Analysis Software (Olympus Soft Imaging Solutions) V2.7.1<br>Rstudio v. 1.4.1717

For manuscripts utilizing custom algorithms or software that are central to the research but not yet described in published literature, software must be made available to editors and reviewers. We strongly encourage code deposition in a community repository (e.g. GitHub). See the Nature Research guidelines for submitting code & software for further information.

Data

Policy information about availability of data

All manuscripts must include a data availability statement. This statement should provide the following information, where applicable:

- Accession codes, unique identifiers, or web links for publicly available datasets
- A list of figures that have associated raw data
- A description of any restrictions on data availability

Microarray data from this publication has been deposited in the National Center for Biotechnology Information Gene Expression Omnibus (GEO) under accession code GSE172426 [https://www.ncbi.nlm.nih.gov/geo/query/acc.cgi?acc=GSE172426]. Raw data associated with Figures can be found in the Supplementary Tables respectively. The quantitative data of this study are available within the paper and its supplementary information files. Source data are provided with this paper for the graphical representations in Figures. 1i, 1j, 1k, 2e, 2f, 2g, 2h, 2i, 2l, 2s, 2t, 3d, 3e, 3f, 4d, 4e, 4j, 4k, 5a, 5b, 5d, 5e, Supplementary Figures. 1d, 1e, 4b, 4c, 5a, 5b. Previously published microarray data that were re-analysed here are available under accession codes GSE87076 (https://www.ncbi.nlm.nih.gov/geo/query/acc.cgi?acc=GSE87076) and GSE63514 (https://www.ncbi.nlm.nih.gov/geo/query/acc.cgi?acc=GSE63514). Uncropped images of the PCR gels and western blots labeled with identifying information are provided as Supplementary Fig. 6 and Supplementary Fig. 7 respectively. All other data supporting the findings of this study are available from the corresponding author on reasonable request.

Field-specific reporting

Please select the one below that is the best fit for your research. If you are not sure, read the appropriate sections before making your selection.

[x] Life sciences [ ] Behavioural & social sciences [ ] Ecological, evolutionary & environmental sciences

For a reference copy of the document with all sections, see nature.com/documents/nr-reporting-summary-flat.pdf

Life sciences study design

All studies must disclose on these points even when the disclosure is negative.

Sample size	Sample size was not predetermined by statistics. The sample size was based on availability of human biopsies and previous experience. Experiments were were performed on n=3 biological replicates except where stated otherwise.
Data exclusions	No data were excluded from the experiments.
Replication	All attempts of replication were sucessful. All graphs and images represent findings from at least two independent repicates, see figure legends. All microarray anaysis represent data from three independent experiments.
Randomization	No specific procedures were carried out for randomization. We mainly used organoids derived from three donors negative for high-risk HPV and respective HPV16 E6E7 integrated organoids.
Blinding	The investigator was blinded for data collection and quantitative analysis.

Reporting for specific materials, systems and methods

We require information from authors about some types of materials, experimental systems and methods used in many studies. Here, indicate whether each material, system or method listed is relevant to your study. If you are not sure if a list item applies to your research, read the appropriate section before selecting a response.

Materials & experimental systems

n/a	Involved in the study	
[ ]	[x]	Antibodies
[ ]	[x]	Eukaryotic cell lines
[x]	[ ]	Palaeontology and archaeology
[x]	[ ]	Animals and other organisms
[ ]	[x]	Human research participants
[x]	[ ]	Clinical data
[x]	[ ]	Dual use research of concern

Methods

n/a	Involved in the study	
[x]	[ ]	ChIP-seq
[ ]	[x]	Flow cytometry
[x]	[ ]	MRI-based neuroimaging

Antibodies

Antibodies used	Mouse-anti-p63 (4A4) (1:200, Abcam, # ab735; Lot GR32772034)<br>Mouse-anti-E-Cadherin (1:100, BD Biosciences, # 610181; Lot 7187865)

Rabbit-anti-Ki67 (SP6) (1:200, Abcam, # ab16667; Lot GR3259333-8)
Rabbit-anti-Cytokeratin 5-Alexa488 (1:300, Abcam, # ab193894; Lot GR3277663-1)
Rabbit-anti-Cytokeratin 8 (1:200, Abcam, # ab59400; Lot GR3201890-4)
Rabbit-anti-Loricrin (1:50, Abcam, # ab85679; Lot GR3262343-1)
Mouse-anti-phospho γH2AX (Ser139) (1:500, Millipore, #05636; Lot NG1951659)
Rabbit-anti-Msh6 (EPR3945) (1:1000, Abcam, # ab92471; Lot GR262215-11)
Rabbit-anti-Mlh1 (EPR3894) (1:10000, Abcam, # ab92312; Lot GR305-730-8)
Mouse-anti-β-Actin (1:10000, Sigma, # 014M4759; Lot 116M4801V)
Mouse-anti-E2F1 (8G9) (1:250, Abcam, # ab 135251; Lot GR260373-16)
Mouse-anti-Rb (4H1) (1:2000, Cell Signaling, # 9309; Lot 9 and 14)
Rabbit-anti-pRb (Ser807/811) (1:1000, Cell Signaling, # 9308; Lot 14)
Mouse-anti-p53 (DO-1) (1:500, Santa Cruz, # sc-126; Lot C0414)
Mouse-anti-Chlamydia Hsp60 (A57-E4) (1:500, Enzo Life Sciences, # ALX-804-071-R100; Lot 03191406 and 1:1000 GeneTex, # GTX25486; Lot 821802599)
Goat-anti-Chlamydia major outer membrane protein (MOMP) (1:500, AbD Serotec # 1990-0804; Lot 120312)
Mouse monoclonal species-specific KK-12 IgG2a Ctr (anti-MOMP) (1:10000, D. Grayston, University of Washington, Seattle, WA, USA)
Donkey-anti-goat Alexa Fluor 488 (1:150, Jackson ImmunoResearch, # 705-545-147; Lot 102410)
Donkey-anti-rabbit-Cy3 (1:150, Jackson ImmunoResearch, # 711-166-152; Lot 101657)
Donkey-anti-mouse Alexa Fluor 647 (1:150, Jackson ImmunoResearch, # 715-605-150; Lot 100906)
Goat anti-mouse-Cy3 (1:150, Dianova, # 115-165-006; Lot 121776)
Donkey-a-goat- Dylight 647 (1:150, Dianova, # 705-605-003; Lot 121791) (1:150)
Donkey-anti-mouse Alexa Fluor 488 (1:150, Dianova, # 715-454-151; Lot 106498)
Sheep-anti-mouse IgG-HRP (1:2000, Amersham Biosciencesa, # NA931; Lot 9451753)
Donkey-anti-rabbit IgG-HRP (1:2000, Amersham Biosciencesa, # NA934; Lot 9451569).

Validation

All antibodies are commercially available and validation experiments for the respective antibodies were performed by the commercial manufacturer and below we provide the respective link for each antibody:
Mouse-anti-p63
https://www.abcam.com/p63-antibody-4a4-ab735.html
Mouse-anti-E-Cadherin
https://www.bdbiosciences.com/en-de/products/reagents/microscopy-imaging-reagents/immunofluorescence-reagents/purified-mouse-anti-e-cadherin.610181
Rabbit-anti-Ki67 (SP6)
https://www.abcam.com/ki67-antibody-sp6-ab16667.html
Rabbit-anti-Cytokeratin 5-Alexa488
https://www.abcam.com/alexa-fluor-488-cytokeratin-5-antibody-ep1601y-ab193894.html
Rabbit-anti-Cytokeratin 8
https://www.abcam.com/cytokeratin-8-antibody-ab59400.html
Rabbit-anti-Loricrin
https://www.abcam.com/loricrin-antibody-ab85679.html
Mouse-anti-phospho γH2AX (Ser139)
https://www.merckmillipore.com/DE/de/product/Anti-phospho-Histone-H2A.X-Ser139-Antibody-clone-JBW301,MM_NF-05-636?ReferrerURL=https%3A%2F%2Fwww.google.com%2F&bd=1
Rabbit-anti-Msh6 (EPR3945)
https://www.abcam.com/nav/primary-antibodies/rabbit-monoclonal-antibodies/msh6-antibody-epr3945-ab92471.html
Rabbit-anti-Mlh1 (EPR3894)
https://www.abcam.com/mlh1-antibody-epr3894-ab92312.html
Mouse-anti-β-Actin
https://www.sigmaaldrich.com/DE/de/product/sigma/a5441
Mouse-anti-E2F1 (8G9)
https://www.abcam.com/E2F1-antibody-8G9-ab135251.html
Mouse-anti-Rb (4H1)
https://www.cellsignal.de/products/primary-antibodies/rb-4h1-mouse-mab/9309
Rabbit-anti-pRb (Ser807/811)
https://www.cellsignal.de/products/primary-antibodies/phospho-rb-ser807-811-antibody/9308
Mouse-anti-p53 (DO-1)
https://www.scbt.com/p/p53-antibody-do-1
Mouse-anti-Chlamydia Hsp60 (A57-E4)
https://www.enzolifesciences.com/ALX-804-071/hsp60-bacterial-monoclonal-antibody-a57-e4/
https://www.genetex.com/Product/Detail/HSP60-antibody-A57-E4/GTX25486
Goat-anti-Chlamydia major outer membrane protein (MOMP)
https://www.bio-rad-antibodies.com/polyclonal/bacterial-chlamydia-trachomatis-momp-antibody-1990-0804.html
Donkey-anti-goat Alexa Fluor 488
https://www.jacksonimmuno.com/catalog/products/705-545-147
Donkey-anti-rabbit-Cy3
https://www.jacksonimmuno.com/catalog/products/711-166-152
Donkey-anti-mouse Alexa Fluor 647
https://www.jacksonimmuno.com/catalog/products/715-605-150
Goat anti-mouse-Cy3

https://www.dianova.com/shop/115-165-006-ziege-igg-anti-maus-igg-fab2-cy3-minx-keine/
Donkey-a-goat- Dylight 647
https://www.dianova.com/en/shop/705-605-003-donkey-igg-anti-goat-igg-hl-alexa-fluor-647-minx-none/
Sheep-anti-mouse IgG-HRP
https://www.citeab.com/antibodies/3288287-na931-1ml-amersham-ecl-mouse-igg-hrp-linked-whole-a
Donkey-anti-rabbit IgG-HRP
https://www.citeab.com/antibodies/3288289-na934-1ml-amersham-ecl-rabbit-igg-hrp-linked-whole

Eukaryotic cell lines

Policy information about cell lines

Cell line source(s)	HeLa cells (ATCC® CCL-2.1); End1 cells (ATCC® CRL-2615); 293T cell (Invitrogen-10938-025). Human keratinocytes containing episomal HPV16 (KC-Epi) and J2-3T3 cell line were a kind gift from Craig Meyers, Department of Microbiology and Immunology, Penn State University School of Medicine, Hershey, Pennsylvania 17033, USA. W12-20850 cells containing HPV16 in the episomal state (W12-Epi) and W12-20831 cells where HPV16 is integrated (W12-Int), a kind gift from Paul F. Lambert, University of Wisconsin-Madison, Madison, WI, USA.
Authentication	HeLa, End1 and 293T cells were ordered as an authenticated cell line from ATCC or Invitrogen respectively. The kindly donated cell lines were not authenticated by us.
Mycoplasma contamination	All cell lines were tested negative for mycoplasma contamination.
Commonly misidentified lines (See ICLAC register)	We used HeLa cells for infectivity assay as they are a standard cell line for confirming C. trachomatis infection.

Human research participants

Policy information about studies involving human research participants

Population characteristics	Tissue biopsies for human ectocervix organoid cultures were obtained from anonymous female donors aged between 35 and 75. Only biopsies of pathologically healthy tissue were used.
Recruitment	Healthy cervical tissue biopsies were obtained from patients undergoing standard surgical procedures for benign gynecological disease at the Department of Gynecology, Charité University Hospital, and August-Viktoria Klinikum, Berlin. Samples were obtained from patients who provided informed consent. The collected samples were tested for HPV status, and samples from different donors were randomly assigned to experiments.
Ethics oversight	Human cervical samples were provided by the Department of Gynecology, Charité University Hospital, and August-Viktoria Klinikum, Berlin. Usage for scientific research was approved by the Ethics Commision of the Charité University Hospital, Berlin, Germany (EA1/059/15); informed consent to use their tissue for scientific usage was obtained from all donors. The study complies with all relevant ethical regulations regarding research involving human participants. Biopsies were sourced from standard surgical procedures for benign gynecological disease. Only anatomically normal cervical tissue were used.

Note that full information on the approval of the study protocol must also be provided in the manuscript.

Flow Cytometry

Plots

Confirm that:

- [x] The axis labels state the marker and fluorochrome used (e.g. CD4-FITC).
- [x] The axis scales are clearly visible. Include numbers along axes only for bottom left plot of group (a 'group' is an analysis of identical markers).
- [x] All plots are contour plots with outliers or pseudocolor plots.
- [x] A numerical value for number of cells or percentage (with statistics) is provided.

Methodology

Sample preparation	Human primary cells were nucleofected with reporter plasmids followed by C. trachomatis infection. At 2h post infection infected and non-infected cells were plated on collagen coated cell culture plates and cultured at 35 °C, 5% CO2 in a humidified incubator.One day after nucleofection cells were washed once with 1x PBS, followed by TrypLE incubation for approximately 10-15 minutes at 37°C. After incubation 5 mL of ADF+++ medium was added and cells were pelleted by centrifugation (5 min at 1000 g; 4°C), the supernatant was discarded and the pellet was resuspended in 500 µL PBS. The suspension was transferred to 75-mm falcon tubes with Cell Strainer Caps (Fisher Scientific) and placed on ice till analysis. Propidium iodide (PI, Sigma) was added to the cells 5 min before analysis
Instrument	FACSymphony™ A5 (BD Biosciences)
Software	FACSDiva software (BD Biosciences) and FlowJo (FlowJo LLC)

Cell population abundance	Only flow cytometric analysis was perfomed, no sorting.
Gating strategy	Cell debris was eliminated from the initial cell population by gating SSC-A/FSC-A. Singlets were discriminated by forward scatter FSC-H/ FSC-W and side scatter SSC-H/SSC-W. Reporter expression (mOrange) was discriminated by b-635LP 670/30-C-A/FSC-A and b-505LP 530/30-E-A/v-450/50-H-A or (mPlum) yg-635LP 670/30-C-A/FSC-A.<br>Boundaries between positive and negative cells was defined by unstained and single stained controls.

☒ Tick this box to confirm that a figure exemplifying the gating strategy is provided in the Supplementary Information.
